# Acid Drinks

**Published:** 1883-06

**Authors:** J. H. Hanaford


### ACID DRINKS.

It is an established principle that cold and hot weather demand clothing and food of a different character, adapted to the varying circumstances. In cold climates and in cold weather the appetite demands, and the comfort of the body justifies, an increased use of carbonaceous food, that the animal heat may be sustained at the vital point of about 78 degrees Fahr. The natural appetite, which is a good guide, demands a change in diet as the spring opens; the disuse of the concentrated foods, the oils, sweets, etc., and the adoption of the succulent products, with those especially containing the sub acids. And, in accordance with this fact, our ever-watchful and kind Provider has given us just the needed medicine foods on the return of warm weather, the berries which are more acid than those appearing later in the season, as all may observe. They are demanded as one means of thinning and purifying the blood, or ridding it of the carbon accumulated during the winter. These acids stimulate the liver to increased activity, removing the waste matter, including that especially from the brain and nerves, which we call "bile," which, in accordance with the same wise and beneficent plan, acts as nature's cathartic, stimulating the bowels, etc., to normal action. It is also true that custom, based on a temporary "craving," demands the use of greens and the like, the prominent feature of which is the acids used—the cooling element. It is unfortunate that in the use of acids we do not select the best, as the natural ones, instead of vinegar, produced by the last or putrefactive stage of the fermentive process, and that in the use of pickles we do not use ripe products, as the beet and the like, instead of the acrid and worthless one-tenth grown cucumber. If we need the acids—as we do—it is far safer to employ them as drinks, as the tartaric acid, the citric and malic, thus avoiding unripe and semi-poisonous fruits. Of the spring medicines I know of none equal to the Acid Phosphate made under the supervision of Professor Horsford, late Rumford Professor at Harvard. It is not merely a medicine, but a food as well, containing, in addition, a pleasant acid—like the "Bread Preparation"—some of the important constituents of the human body. It may be used as a substitute for lemons in drinks, or as a medicine in bilious affections, but is still more efficacious in its action on the brain and nerves, reaching cases of "nervous prostration," so prevalent. It is believed that it may well displace the cathartics, the fashionable "bitters" —often decidedly intoxicating—as well as the "tonics" and stimulants which do so much harm by undue forcing of the system, to be succeeded by a reaction, instead of nourishing the body.

J. H. HANAFORD, M. D.

